# Navigating OCD During Pregnancy: A Case Study and Therapeutic Insights

**DOI:** 10.1192/j.eurpsy.2025.2387

**Published:** 2025-08-26

**Authors:** B. Arribas-Simon, O. Martin-Santiago, C. Alario-Ruiz, O. Segurado-Martin, M. J. Mateos-Sexmero, P. Pando-Fernandez, P. Martinez-Gimeno, B. Rodriguez-Rodriguez, N. Navarro-Barriga, P. Andres-Olivera, M. Calvo-Valcarcel, M. Andreo-Vidal, F.-J. Gonzalez-Zapatero

**Affiliations:** 1 Psychiatry, Hospital Clinico Universitario, Valladolid; 2 Psychiatry, CAUSA, Salamanca, Spain

## Abstract

**Introduction:**

Obsessive-Compulsive Disorder (OCD) during pregnancy can worsen due to hormonal changes, psychological stress, and concerns about the baby’s health. It presents unique challenges for diagnosis and treatment, balancing the mother’s mental health with fetal safety. This case focuses on a woman who developed OCD in her third trimester, emphasizing the challenges in managing the condition.

**Objectives:**

- To describe the impact and progression of OCD during pregnancy.
- To assess the effectiveness of Cognitive Behavioral Therapy (CBT) and evaluate pharmacological options.
- To analyze the risks and benefits of managing OCD therapeutically in pregnant women.

**Methods:**

A clinical case of a 32-year-old woman at 28 weeks of gestation, with newly diagnosed OCD, is presented. Symptoms began in the second trimester with intrusive thoughts about harming her baby and compulsive checking and cleaning behaviors. The patient was treated with CBT, and SSRIs were considered due to symptom severity. Follow-up continued through pregnancy until delivery.

**Results:**

CBT led to a significant reduction in compulsions and improved management of obsessive thoughts. However, moderate symptoms persisted, leading to consideration of SSRIs, which were ultimately avoided due to concerns about side effects. The patient’s delivery was uncomplicated, and continued CBT postpartum resulted in significant improvement.

**Conclusions:**

This case illustrates the complexity of treating OCD during pregnancy, where hormonal changes and concerns about fetal health can exacerbate symptoms. Early intervention with CBT can be effective, and treatment decisions must carefully balance maternal and fetal well-being.

**Disclosure of Interest:**

None Declared

